# Genetic Basis for Dosage Sensitivity in Arabidopsis thaliana

**DOI:** 10.1371/journal.pgen.0030070

**Published:** 2007-04-27

**Authors:** Isabelle M Henry, Brian P Dilkes, Luca Comai

**Affiliations:** Department of Biology, University of Washington, Seattle, Washington, United States of America; University of Wisconsin, United States of America

## Abstract

Aneuploidy, the relative excess or deficiency of specific chromosome types, results in gene dosage imbalance. Plants can produce viable and fertile aneuploid individuals, while most animal aneuploids are inviable or developmentally abnormal. The swarms of aneuploid progeny produced by *Arabidopsis* triploids constitute an excellent model to investigate the mechanisms governing dosage sensitivity and aneuploid syndromes. Indeed, genotype alters the frequency of aneuploid types within these swarms. Recombinant inbred lines that were derived from a triploid hybrid segregated into diploid and tetraploid individuals. In these recombinant inbred lines, a single locus, which we call *SENSITIVE TO DOSAGE IMBALANCE (SDI),* exhibited segregation distortion in the tetraploid subpopulation only. Recent progress in quantitative genotyping now allows molecular karyotyping and genetic analysis of aneuploid populations. In this study, we investigated the causes of the ploidy-specific distortion at *SDI*. Allele frequency was distorted in the aneuploid swarms produced by the triploid hybrid. We developed a simple quantitative measure for aneuploidy lethality and using this measure demonstrated that distortion was greatest in the aneuploids facing the strongest viability selection. When triploids were crossed to euploids, the progeny, which lack severe aneuploids, exhibited no distortion at *SDI*. Genetic characterization of *SDI* in the aneuploid swarm identified a mechanism governing aneuploid survival, perhaps by buffering the effects of dosage imbalance. As such, *SDI* could increase the likelihood of retaining genomic rearrangements such as segmental duplications. Additionally, in species where triploids are fertile, aneuploid survival would facilitate gene flow between diploid and tetraploid populations via a triploid bridge and prevent polyploid speciation. Our results demonstrate that positional cloning of loci affecting traits in populations containing ploidy and chromosome number variants is now feasible using quantitative genotyping approaches.

## Author Summary

Each eukaryotic genome is subdivided into a specific number of chromosome types, which in turn are present in a characteristic number of copies, usually the same for all chromosomes. In the condition called aneuploidy, copy number differs among chromosome types, disrupting their balance and that of their encoded factors. As a result, aneuploidy is associated with developmental defects and death. For example, most types of human aneuploids are unviable: the only autosomal aneuploidy compatible with protracted survival, Down syndrome, is caused by the presence of three copies, instead of two, of the very small Chromosome 21. In plants, aneuploidy is more common and less deleterious. This suggests that plants can more easily tolerate the effects of aneuploidy and can be used to investigate them. Here, we used the model plant A*rabidopsis thaliana* to produce and investigate populations of aneuploid individuals. By comparing genetically distinct aneuploid populations, we identified a chromosomal region that is associated with greater aneuploid survival. Characterizing the genetic mechanism modulating the response to changes in chromosomal dosage and aneuploid survival will help understand how genome organization affects biological processes and why aneuploidy results in such severe developmental defects.

## Introduction

Most eukaryotic genomes maintain genes in a one-to-one relationship by their syntenic organization on chromosomes. This normal stoichiometry between chromosomes of a set can sometimes be disrupted, resulting in altered dosage of both genes and their encoded products. Such disruptions can arise via the nondisjunction of chromatids and chromosomes during mitosis and meiosis and result in uneven chromosome numbers, a condition called aneuploidy. Trisomy, the most common form of viable aneuploidy is characterized by the presence of one extra chromosome in an otherwise diploid background. The observation of stereotypical phenotypes for trisomics of each chromosome type illustrated that genetic factors are sensitive to dosage [1–5]. Indeed, the proper functioning of cells and organisms relies on molecular complexes, which require a delicate balance between components for proper operation [6]. Even a slight departure from this balance can have dramatic phenotypic or developmental consequences [6,7] as exemplified by the many haplo-insufficient genes identified in human as tumor suppressors [8] and as essential or regulatory genes in yeast [7,9] and *Drosophila* [10,11]. In aneuploids, where dosage variations affect whole chromosomes rather than single genes, the consequences can be severe when the copy numbers of many dosage-sensitive genes are altered at once. Therefore, an alteration of gene dosage such as it occurs in aneuploids typically has unfavorable consequences.

Interestingly, aneuploidy is not always deleterious and can be persistent. For example, aneuploid cells are normally found in certain tissues such as the brain and the placenta, where they appear to play a functional role [12–15]. Aneuploidy has been associated with invasive cancer [16,17] and controversially proposed to play a causal role in malignancy [18]. Although cancer is obviously deleterious to the affected organism, somatic selection of aneuploid sectors underscores the fact that dosage imbalance can be advantageous to cells. Finally, aneuploid individuals are common in plants and in yeast and provide a pool of phenotypic variation not present in the euploid population. In specific conditions, these phenotypes can be advantageous, and the corresponding aneuploid karyotypes selected. Such successful aneuploids have been observed both in nature and in industry [19–22]. Thus, although dramatic alterations of phenotype are associated with aneuploidy, this condition can be compatible with efficient function and even fitness.

There is also tremendous variation for aneuploidy tolerance between different organisms. For reasons that remain unknown, plants are generally less sensitive than animals to the type of dosage imbalance caused by aneuploidy [17]. Indeed, in humans, most aneuploidies are embryo-lethal, and the few that are viable are associated with severe developmental defects [23]. In contrast, trisomics of all chromosome types as well as more complex aneuploid types have been described in several plant species [23–27]. There is also considerable variation between plant species in the degree of lethality caused by dosage imbalance. For example, the progeny produced by triploids of different plant species vary in the extent and frequency of aneuploidy. During triploid meiosis, three sets of chromosomes must be allocated to two poles, producing mostly aneuploid gametes. The progeny produced by such gametes should consist of a swarm of aneuploid types ranging from near diploid to near tetraploid. Such a swarm is produced by triploids of certain species, such as A. thaliana [24], but triploids of other species fail to produce such a range of aneuploids generating instead mostly diploids and near diploids [3,23,28,29]. Finally, sensitivity to aneuploidy can differ between varieties of the same species. One such case was reported in tomato in which cherry tomato produced aneuploids with an average of one more extra chromosome than those of a large-fruited tomato variety [30]. Similar observations were made in barley in which the aneuploids produced by a wild variety carried a higher number of extra chromosomes, were more vigorous, and exhibited higher fertility than the aneuploids produced by a triploid of a cultivated variety [31,32]. In A. thaliana, comparison of identical trisomics of the Columbia (Col-0) and Landsberg erecta ecotypes uncovered differences in fertility and transmission rate of the trisomic chromosome [5,33–35]. A detailed genetic characterization of natural variation for tolerance to aneuploidy and cloning of the responsible loci have so far not been possible. Recent technological advances in *Arabidopsis* that combine molecular karyotyping and quantitative genotyping now allow quantitative genetic analysis of aneuploid populations [36]. Here, we report the first step towards positional cloning of a locus affecting aneuploid survival in *Arabidopsis.*

**Figure 1:**
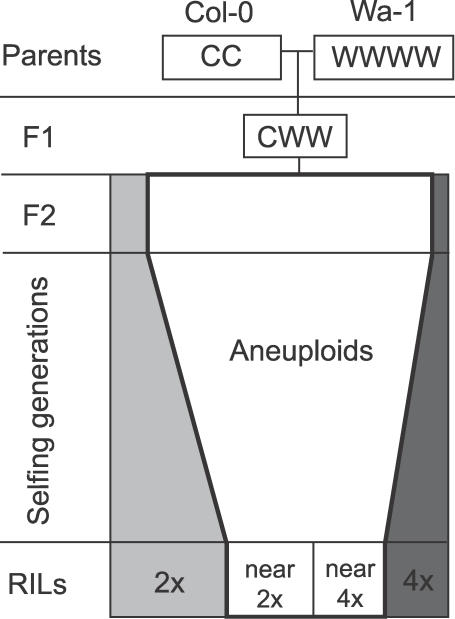
Diploid, Tetraploid, and Aneuploid Individuals in the Progeny of a Triploid The proportions of diploid (light gray), tetraploid (dark gray), and aneuploid (white) individuals represent those observed in the CWW *F*_2_ population and the Col-0 × Wa-1 RILs [24]. The progressive decline of aneuploidy towards euploidy during the selfing generations is extrapolated.

We previously investigated the effect of genotype on the rate of aneuploidy production by comparing the karyotype swarms in the progeny of two triploids of A. thaliana. One genotype, the CCC triploid, was produced from a cross between diploid Col-0 and its synthetically derived tetraploid (4x-Col). The other, the CWW triploid, was produced from a cross between diploid Col-0 and the naturally occurring tetraploid Warschau (Wa-1). We demonstrated that both of these triploids were fertile and produced a swarm of aneuploid progeny [24]. Genotype influenced both fertility of the triploids and the composition and performance of their aneuploid swarms. Additionally, recombinant inbred lines (RILs) produced from the progeny of a CWW triploid [37] resolved into two cohorts of near-diploid and near-tetraploid genome contents (Figure 1). Genetic analysis of these RILs identified transmission distortion at genetic markers on Chromosome 1 in the near-tetraploid but not the near-diploid lines [24]. In the present report, we investigated the genetic mechanisms responsible for the ploidy-dependent selection of this locus, which we call *SENSITIVE TO DOSAGE IMBALANCE (SDI)*. Our results demonstrate that transmission distortion favoring the Wa-1 allele of *SDI* occurs in genome content classes containing the most severe aneuploids, produced by selfing a triploid. This indicates a role for *SDI* in aneuploidy survival, possibly by buffering the dosage-related challenges associated with aneuploidy. Potential mechanisms consistent with these observations and their evolutionary implications are discussed.

## Results

Genetic analysis of the Col-0 by Wa-1 RILs identified a locus, *SDI,* linked to markers nga280 and MN1.2 on Chromosome 1. The high percentage of the Wa-1 allele at *SDI* in the near-tetraploid RILs could result from selection at different steps. For example, selection of the Wa-1 allele could occur via a role in tetraploid maintenance or survival. Indeed, previous analyses demonstrated that tetraploidy is unstable and that tetraploid individuals often produce aneuploids [36]. Alternatively, selection could occur in the early generations by modulating the sensitivity of plants to aneuploidy. Consistent with this alternative, a higher percentage of aneuploids were found in the near-tetraploid lines than in the near-diploid lines (Figure 1) [24]. A protracted aneuploid phase might result in selection for alleles that reduce the deleterious effects of aneuploidy. To test these two hypotheses and identify a mechanism for the ploidy-dependent selection at *SDI,* we analyzed the inheritance of markers linked to *SDI* in three other populations.

### The *SDI* Locus Is Not Selected in a Tetraploid Population

**Figure 2:**
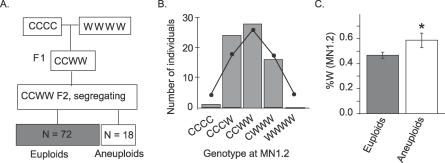
Analysis of Allele Transmission at *SDI* in a Tetraploid Population (A) Generation of the CCWW *F*_2_ population. (B) Expected (line) and observed (bars) genotype frequencies at the MN1.2 marker in the CCWW *F*_2_ population. (C) The mean percentage of Wa-1 allele at MN1.2 was significantly higher in the aneuploid than in the euploid individuals (*t*-test *p*-value = 0.0396). Standard errors are indicated.

We produced an *F*_2_ family from tetraploid CCWW plants (Figure 2A). Ninety CCWW *F*_2_ individuals were genotyped at 11 markers, including nga280 and the linked MN1.2. No markers exhibited transmission ratio distortion in this population (Figure 2B). Thus, the polymorphism at *SDI* is unlikely to be critical for the survival of tetraploids.

### The *SDI* Locus Is Selected in Aneuploid Individuals

As expected from our previous studies of tetraploids [36], several aneuploid individuals were identified among this CCWW *F*_2_ population (Figure 2A). To investigate whether the *SDI* allele from Wa-1 was selected in the aneuploid individuals in this *F*_2_ population, the percentage of the Wa-1 allele in the aneuploid and tetraploid subpopulations were compared. Of the 11 markers tested, only MN1.2 (but not nga280) exhibited a significant difference between the two subpopulations. A higher percentage of the Wa-1 allele was present in the aneuploids than in the euploid tetraploid *F*_2_ progeny (*t*-test *p*-value = 0.0396) (Figure 2C).

**Figure 3:**
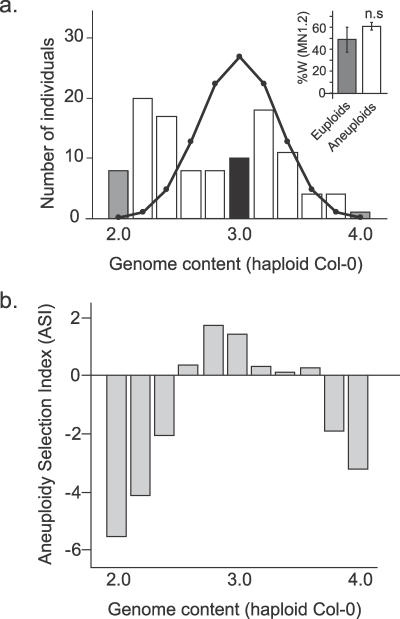
Calculation of the ASI for Each Genome Content Class in the CWW *F*_2_ Population Genome content is expressed as multiples of the nuclear genome content of haploid Col-0. (A) The number of individuals in each genome content class observed in the CWW *F*_2_ population (bars) was compared to the expected numbers based on random assortment of chromosomes at meiosis and absence of selection (line). Euploid classes (diploids and tetraploids) are represented by gray bars, while aneuploid classes are represented by white bars. Inset: the mean percentage of Wa-1 allele at MN1.2 was higher in the aneuploid than in the euploid individuals but the difference was not significant (t-test *p*-value = 0.382). Individuals with a genome content of 3.0 (black bars) were excluded from this analysis, because it was not possible to determine their exact karyotype and whether they were true triploids or aneuploids with a genome content consistent with 15 chromosomes. (B) ASI values for each genome content class. Negative values indicate that a class is observed more often than expected, while positive values indicate that a class is observed less often than expected.

The previously characterized populations derived by selfing of the CWW triploid [24] were tested for selection at *SDI*. In both the CWW *F*_2_ population and the near-diploid RILs, the percentage of Wa-1 allele at MN1.2 was, on average, higher in the aneuploid individuals than in the euploid individuals (Figure 3A, inset). This trend was weak and not significant (*t*-test *p*-value = 0.38 and 0.34, respectively). A shortcoming of grouping all CWW-produced aneuploids is that differences in the severity of aneuploidy and thus differences in selection for aneuploidy tolerance were not accounted for.

### Karyotype Frequency as a Proxy for Aneuploidy Lethality

To investigate the relationship between selection of the Wa-1 allele and aneuploidy severity, a quantitative measure of karyotype-dependent selection was developed. As previously reported, the CWW *F*_2_ is a complex swarm of aneuploids of various karyotypes [24]. This swarm does not match the predicted outcome of triploid meiosis, presumably due to lethality differentially affecting these karyotypes [24]. The expected frequency of each genome content class was calculated previously (see Figure 2C in [24]). For each genome content class, the ratio of expected-to-observed frequencies (Figure 3A) was used to calculate the aneuploidy selection index (ASI) (see Material and Methods for details) (Figure 3B). Negative and positive values for ASI indicated overrepresentation and underrepresentation of a class relative to the expected frequency, respectively.

**Figure 4:**
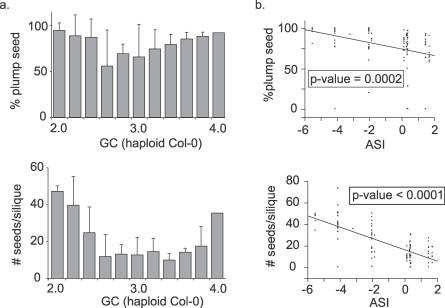
Biological Relevance of the ASI (A) For each individual in the CWW *F*_2_ population, total number of seed (top panel) and percentage of plump seed (bottom panel) were recorded. Data for all individuals in the same genome content class were pooled, and mean values were calculated (bars). Standard errors are indicated. (B) The relationship between ASI and total seed number (top panel) or percentage of plump seed (bottom panel) was estimated by regression analysis. Both regressions were significant, and *p*-values are indicated.

To test the biological significance of the ASI, we examined the relationship between ASI and seed production in the CWW *F*_2_ population. Both the percentage of plump seed (as an estimate of seed viability) and the total number of seed produced by each of the CWW *F*_2_ individuals were recorded (Figure 4A). Their relationship with ASI was determined by regression analyses (Figure 4B). Both regressions were highly significant (*p*-value = 0.0002 for seed viability and <0.0001 for seed counts). This demonstrated that ASI is a biologically relevant measure correlated with the viability selection acting upon aneuploid classes and predicts the strength of viability selection in the next generation. Thus, ASI represents an excellent early indicator of karyotype-modulated viability selection.

### Genotype at *SDI* Is Associated with the Strength of Selection in the Triploid Swarm

**Figure 5:**
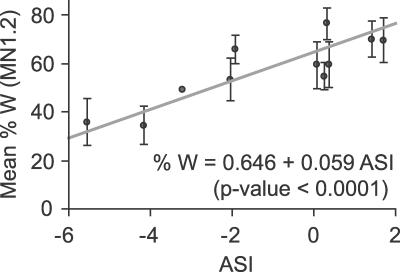
Relationship between Marker Genotype at MN1.2 and ASI in the CWW *F*_2_ Population For each ASI value, the mean percentage of Wa-1 allele (gray dots) and corresponding standard errors (bars) are presented. No standard error could be calculated for classes containing only one individual. A regression curve was calculated using each individual independently, and the regression was significant (*p*-value < 0.0001, *R* ^2^ = 0.16).

To test whether the Wa-1 allele at *SDI* could be linked to increased survival of aneuploid individuals, the relationship between marker genotype and ASI was investigated by regression analysis. The percentage of Wa-1 allele at three markers, all located at the bottom of Chromosome 1 were significantly associated with ASI (Table 1). The most significant association between genotype and ASI was found at MN1.2 (Figure 5). This association was consistent with a role for *SDI* in modulating the viability of aneuploids.

**Table 1:**
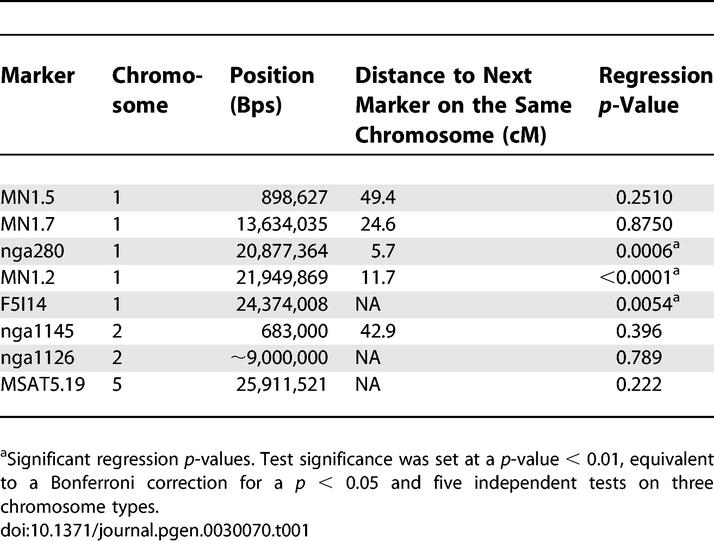
Analysis of the Relationship between Marker Genotype and ASI in the CWW *F*_2_ Population

Unfortunately, because there is no diploid of Wa-1, it was not possible to perform a similar analysis on the progeny of a CCW triploid, which could have controlled for potential effects of preferential pairing or segregation. It is possible that an unidentified meiotic mechanism affected chromosome segregation such that it would be responsible for the effect observed at *SDI*. We have observed that the percentage Wa-1 allele at *SDI* increases with genome content (regression *p*-value = 0.0006, *r* ^2^ = 0.12). Yet, when the effects of genome content and ASI were tested simultaneously, only the effect of ASI (*p*-value = 0.0096) remained significant while that of genome content (*p*-value = 0.41) did not. This suggested that the apparent increase of percentage Wa-1 allele with genome content was due to the correlation between ASI and genome content (regression *p*-value < 0.0001, *r* ^2^ = 0.44) and not due to an overall increased percentage Wa-1 allele in disomic gametes.

The percentage Wa-1 allele at MN1.2 was lower than expected in the diploid individuals and aneuploids of low genome content (Figure 5). This observation was unexpected but appears to fit within a genome-wide phenomenon. In both the CWW *F*_2_ and RIL populations, the percentage Wa-1 allele was on average lower than the expected 66% throughout the genome [37]. Although we do not have an explanation for this observation, marker MN1.2 is not unusual in this respect.

The association between genotype at MN1.2 and karyotype was also analyzed using the progeny of pseudo-backcrosses (pBC) involving the CWW triploid. In these populations, one of the two parents is a CWW triploid while the other parent is either diploid Col-0 or its tetraploid derivative (4x-Col). ASI values for each chromosome content class were calculated separately for each of the four pBC populations: CWW × Col-0; CWW × 4x-Col; Col-0 × CWW; 4x-Col × CWW. An association between ASI and marker genotype was again tested by regression. As observed in the CWW *F*_2_ aneuploid swarm, the percentage of Wa-1 allele at MN1.2 increased with the ASI but the regression was not significant for any of the four populations studied (*p*-values between 0.23 and 0.92). Thus, selection for *SDI* in the progeny of a triploid was only visible in the context of a selfed triploid, where zygotes can be more severely imbalanced and where aneuploidy can have both maternal and paternal origin.

## Discussion

Previously, ploidy-dependent transmission distortion was found at the *SDI* locus in a recombinant inbred population derived from a triploid produced by crossing Col-0 and the naturally collected tetraploid ecotype Wa-1 [24]. To investigate the mechanisms behind the selection of the Wa-1 allele at *SDI* in these RILs, we determined when this selection could have occurred during the derivation of the RILs (Figure 1). First, we demonstrated that allele frequencies at *SDI* were not distorted in tetraploid populations segregating for the Col-0 and Wa-1 alleles, suggesting that the Wa-1 allele of *SDI* was not required for tetraploid maintenance (Figure 2). Markers linked to *SDI* were, however, selected in the aneuploid individuals produced by the CCWW tetraploids (Figure 2C) suggestive of a role for *SDI* in aneuploid viability. To further test this hypothesis, selection at *SDI* was investigated in the aneuploid swarm produced by the selfed CWW triploid. In this population, selection at *SDI* was associated with a quantitative measure of aneuploid survival (Figure 5). Specifically, the Wa-1 allele was strongly selected in those aneuploid genome content classes that exhibited the strongest viability selection (Figures 3 and 5). Selection against aneuploidy results from dosage imbalances affecting cellular functions required either in the gametes, at fertilization or in the fertilization products. We therefore named this locus *SENSITIVE TO DOSAGE IMBALANCE* or *SDI*.

Previously, we identified several other differences in the response of the Col-0 and Wa-1 genomes to triploidy and aneuploidy. Genotype influenced both fertility of the triploid and the composition and performance of the aneuploid swarm produced in the immediate filial generation following triploidy [24]. The CWW triploid produced a higher percentage of live seed and more triploid and aneuploid progeny than the CCC triploid [24]. We also previously observed that the natural tetraploid Wa-1 produced more viable aneuploid individuals than the synthetic tetraploid Col-0 [36]. These results are consistent with our interpretation that the Wa-1 allele at *SDI* improves aneuploid survival.

### Could the Wa-1 Allele at *SDI* Rescue Extreme Aneuploids?

**Figure 6:**
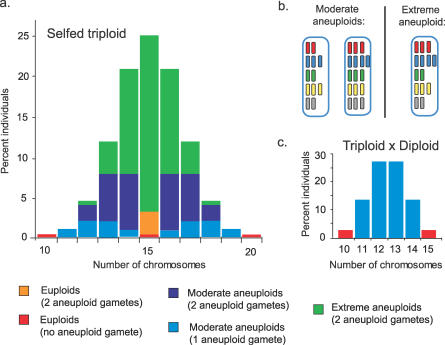
Different Aneuploid Types Found in the Progeny of Triploids (A) The different types of individuals expected in the progeny of a selfed triploid depending on their karyotype and on the karyotype of the gametes that produced them. (B) In moderate aneuploids, the difference in number of copies of different chromosome types is maximum 1. In extreme aneuploids, some chromosome types are present in two (or more) additional copies than other chromosome types. (C) In the progeny of a cross between triploid and diploid individuals, only two types of individuals are found: euploids produced by euploid gametes or aneuploids produced from an aneuploid gamete and a euploid gamete.

The percentage of Wa-1 allele at *SDI* increased with our measure of aneuploidy selection in the CWW *F*_2_ population. Thus, selection at *SDI* in the CWW *F*_2_ was karyotype-dependent. Yet, karyotype-dependent selection at *SDI* was not significant in the progeny of the pBC. Comparing the theoretical population of aneuploids produced by a selfed triploid to those produced in the pBC suggests possible explanations for this observation (Figure 6). The pBC populations are exclusively composed of moderate aneuploid (dosage deviation of no more than one chromosome, light blue) and euploid (red) individuals. These individuals are also present in the triploid *F*_2_ distribution, where they only represent a minor proportion (Figure 6A versus 6C). More extreme aneuploid individuals (in green in Figure 6), containing two copies of some chromosome types and four copies of others can be formed from a selfed triploid when aneuploid gametes carrying extra copies of the same chromosome types fertilize each other. In fact, this class of aneuploids constitutes the majority of the possible CWW *F*_2_ karyotypes and cannot be produced in pBCs, where one parent contributes only euploid gametes (Figure 6). The presence of these extreme aneuploids is supported by the fact that individuals with extreme phenotypes (e.g., extreme dwarfism or complete sterility) were observed in the CWW *F*_2_ but not in the pBCs (unpublished data), and that the genome content classes with higher ASI values are those with the highest predicted frequency of severe aneuploids. The theoretical proportion of these severe aneuploids increases rapidly with increasing chromosome number. For example, triploids with five chromosome types are expected to produce 56% severe aneuploids. On the other hand, triploid individuals with ten chromosome types, such as maize, are expected to produce 89% severe aneuploid progeny. If the severe aneuploid types are more strongly selected against, one would therefore expect that seed yield and viability would be lower in triploids of species with higher chromosome number.

The differences in karyotype distributions between the progeny of a selfed triploid and those from a pBC are not limited to the presence or absence of the extreme aneuploid class. Also present in the triploid *F*_2_ but absent from the pBC, is a second class of moderate aneuploids: those formed following the fusion of two (instead of one) aneuploid gametes (Figure 6A, dark blue). These make up the second largest group of expected progeny in the selfed triploid. In aneuploid gametes, the production of dosage-sensitive factors needed for the success of fertilization and early development is compromised. In a fertilization event in which both gametes are aneuploid, the inappropriate production of factors such as those responsible for interploidy seed failure and the endosperm dosage factors [38] are most likely to result in seed failure. Finally, in a selfed triploid opportunities for selection at the gametophyte generation occur in both pollen and ovules. This doubles the impact of selection on the haploid generation as compared to the pBCs. Consistent with the association of *SDI* selection with aneuploidy severity in the CWW *F*_2_, the highest percentages of Wa-1 allele at *SDI* are associated with the genome content classes that are predicted to contain the highest percentage of severe aneuploids (genome content classes 2.8, 3.0, and 3.2).

As argued above, selection against severe aneuploids may be an important determinant of genome content distribution in triploid progeny. Our data suggest that other processes also contribute to it. If selection against severe aneuploids was the only driving force, one would expect a completely symmetrical bimodal distribution (encompassing the red, orange, and light blue individuals in Figure 6). The observed distribution (Figure 3A) is bimodal but not symmetrical. It has been hypothesized that carrying an excess of several types of chromosomes (such as in genome content classes 2.6 or 2.8) is more deleterious than carrying only one chromosome in excess (genome content classes 2.2 or 3.2). This hypothesis is based on the observation that most regulatory interactions are negative [10]. Therefore, increasing the number of copies at more loci (or stated differently, having a minority of loci in relative deficiency) should increase the probability of negatively affecting loci involved in crucial cellular or developmental processes and that are not located on the additional chromosome copies [10]. Our results agree with this hypothesis: in each half of the distribution, individuals with lower genome contents are more common than individuals with higher genome content (Figure 3A).

### What Are the Evolutionary Consequences of Surviving Dosage Imbalance?

We have shown that the naturally collected tetraploid ecotype Wa-1 produced aneuploid individuals more often than Col-0 [36]. In addition, an allele from Wa-1 is associated with the survival of severe aneuploid individuals (Figures 2 and 5). Considering the obvious negative consequences of aneuploidy, is there a counterbalancing advantage that could justify the persistence of such a trait? Persistent aneuploidy has been reported in several specific situations where the aneuploid phenotype confers a selective advantage relative to the diploid phenotype. For example, segmental aneuploidy is frequent in yeast deletion mutants [39] and can confer a growth advantage to the aneuploid cells compared to the euploid ones [39]. In humans, aneuploid cells have recently been found to be an integral part of the functional pool of neurons and in placental trophoblasts, consistent with a functional role for aneuploidy in these contexts [12–15]. In plants, it is believed that aneuploidy can play a role in speciation and phenotype evolution [40]. Additionally, aneuploidy is very frequent in polyploid populations [2,40] as well as in the process of polyploid formation through the triploid bridge in species for which triploids are readily produced and are fertile [24,41]. In neopolyploids, aneuploidy may contribute to phenotypic variability [40] and become fixed through selection for advantageous karyotypes. An allele associated with increased tolerance to dosage imbalance would therefore increase the probability that advantageous karyotypes arise and reproduce successfully. In addition, it would increase the fertility of triploids produced from unreduced gametes or interploidy hybridization. This would enhance gene flow between diploid and polyploid subpopulations via a triploid bridge and aneuploid swarms and allow the sharing of alleles arising in either population. Such recurrent triploid formation between diploid and polyploid populations and repeated formation of polyploid derivatives would therefore hinder polyploid speciation and may explain why multiple karyotypes are often cataloged as a single species based on shared ecological and morphological traits.

### What Mechanism(s) Underlie the Action of *SDI?*

The fact that selection acting on *SDI* is associated with the presence of extreme aneuploids suggests that *SDI* acts to buffer the effects of dosage imbalance. This buffering could enhance the survival of aneuploid gametes or that of the fertilization products, either the zygote itself or the endosperm and could be mediated through a number of mechanisms.

Selection at *SDI* could stem directly from specific dosage effects on one or a few dosage-sensitive genes. Aneuploidy affects the regulation of genes located both on the varied chromosome and on the rest of the genome [17,42–45]. The intensity of these effects varies from gene to gene and can be positive or negative, illustrating the complexity of the regulatory networks [10,17,44,46]. Observations in maize and *Drosophila* have led to the idea of a “dosage-regulatory hierarchy,” in which the expression of a given gene might be regulated by several dosage-dependent regulators, which in turn are involved in the regulation of several target genes [10]. This hypothesis would be consistent with the possibility that selection at *SDI* stems from the misregulation of a specific dosage-sensitive gene linked to *SDI*.

Karyotype-dependent selection at *SDI* could also originate from a genome-wide effect mediated by *SDI*. Changes in chromosome number affect overall genome maintenance, function, and regulation [47,48]. For example, polyploidy results in variation in epigenetic regulation, as demonstrated by ploidy-sensitive gene silencing and paramutation in *Arabidopsis* [48–50]. Epigenetic silencing in polyploids and aneuploids may result directly from dosage imbalance. This idea is supported by our understanding of the mechanisms underlying dosage compensation in flies, mammals, and worms, which all rely on chromatin remodeling [51]. In plants, trisomy dependent epigenetic instability has been reported for a transgenic locus in tobacco [52,53]. Similarly, cancerous cells are associated with both aneuploidy and epigenetic modifications [17,54]. In addition, meiotic silencing of unpaired DNA has been demonstrated in Neurospora crassa [55], in X-chromosome imprinting in C. elegans [56], and in sex chromosome inactivation in mammals [57]. It is possible that the presence of unpaired chromosomes during triploid or aneuploid meiosis has similar consequences. Thus, it is possible that the *SDI* locus encodes a regulator mediating a genome-wide epigenetic response to dosage imbalance. The possible involvement of *SDI* in epigenetic modifications of the genome or in its regulation is an attractive hypothesis that the recent publication of the complete methylome of A. thaliana [58,59], natural variation in A. thaliana methylation level [60,61], and our ability to detect and karyotype aneuploid individuals [36] will help address.

In conclusion, we have established a quantitative measure for aneuploid survival. Using this trait, we have demonstrated the feasibility of genetic mapping in aneuploids and associated a locus to the variation in aneuploid survival observed in *Arabidopsis.* Characterization of the gene(s) responsible for *SDI* should facilitate a better understanding of the mechanisms governing the sensitivity to dosage imbalance and aneuploid syndromes.

## Materials and Methods

### Plant growth conditions, lines, and crosses.

All plants were grown on soil (Sunshine Professional Peat-Lite mix 4, SunGro Horticulture, http://www.sungro.com) in a growth room lit by fluorescent lamps (Model TL80; Philips, http://www.lighting.philips.com) at 22 ± 3 °C with a 16 h:8 h light:dark photoperiod or in a greenhouse at similar temperatures and light regimes, with supplemental light provided by sodium lamp illumination as required.

Tetraploid lines were described previously [24]. Col-0 represents the diploid ecotype Columbia, 4x-Col represents tetraploidized Col-0, and Wa-1 represents the naturally occurring tetraploid ecotype Warschau-1 (CS6885). C and W refer to basic genomes or alleles of Col-0 and Wa-1, respectively. The CCWW *F*_2_ population (*n* = 90) was obtained by crossing 4x-Col as the seed parent to Wa-1 and allowing three *F*_1_ individuals to self pollinate. The Col-0 × Wa-1 RILs described by Schiff and coworkers [37] were a kind gift from Shauna Somerville (Carnegie Institution, Stanford University). The CWW triploid plants were generated by crossing Col-0 as the seed parent to Wa-1. The CWW *F*_2_ population (*n* = 109) was generated as described [24]. In order to reduce the complexity of the aneuploid swarm produced by a triploid, pBC populations were generated by crossing CWW triploids to either diploid Col-0 or tetraploid 4x-Col, in both directions [36]. The four types of pBC populations and the number of individuals analyzed in the context of this report were as follows: Col-0 × CWW (*n* = 80), CWW × Col-0 (*n* = 102), 4x-Col × CWW (*n* = 33), CWW × 4x-Col (*n* = 47).

### Phenotypic analysis of seed production.

Single siliques were harvested into individual tubes, and all the seeds from each fruit were counted using a dissecting microscope. To estimate seed viability, seeds were characterized as “plump” if they contained a visible embryo structure at least 20% the size of wild-type seed or “shriveled” if they did not. On average, five individual siliques were counted for each individual. Mean values for each group of plants were compared pair-wise using Student's *t*-test and *p*-values < 0.05 were considered significant.

### Flow cytometric determination of genome content.

All individuals in the pBC, the CWW *F*_2_, and the RIL populations were analyzed for genome content as previously described [24,36]. Briefly, control A. thaliana samples of known genome content were run before, between, and after experimental samples and used to create a standard curve, allowing us to determine the genome content of our experimental samples. Individuals were categorized by genome content values expressed as a multiple of the haploid genome content of Col-0. On this scale, a 2.0 corresponds to a diploid individual, and a 4.0 corresponds to a tetraploid individual. The number of categories was chosen based on how many chromosome number classes were expected (four classes between diploidy and triploidy as well as four classes between triploidy and tetraploidy). We have previously shown that this method is accurate and precise for A. thaliana aneuploids by comparing our flow results to complete karyotypes (*r* ^2^ = 0.983 in [36]).

### Quantitative genotyping and karyotyping.

Quantitative genotyping was performed as previously described [36]. Different populations were genotyped at different markers. The progeny of the pBCs were genotyped at all 12 markers previously described [36], and the data were used to infer the complete karyotype of each individual [36]. The CCWW *F*_2_ individuals were genotyped at eight of those 12 markers located on four of the five chromosome types, namely MN1.5, MN1.6, MN1.7, MN1.2, nga1126, nga1145, MN4.2, and MSAT5.19 as well as at the three additional markers nga280, F5I14, and nga692 all located on Chromosome 1. The CWW *F*_2_ individuals were genotyped at MN1.5, MN1.7, MN1.2, nga280, F5I14, nga1126, nga1145, and MSAT5.19 located on three of the five chromosome types. Finally one marker, MN1.2, located approximately 5.7 cM distal to the centromere from nga280, was added to the Col-0 × Wa-1 RILs genotype data [24,37]. Selection at MN1.2 in the near-tetraploid RILs was evaluated according to previous protocols [24] and was identical to that of nga280. In the near-diploid population, the percentage of Wa-1 allele at MN1.2 was slightly higher than at nga280. As a result, comparisons between diploid and tetraploid RILs were not significant after a Bonferroni correction for 11 independent tests but a strong trend was evident (Fisher Exact test *p*-value = 0.009). MN1.2 spans a deletion polymorphism between Col-0 and Wa-1, while nga280 amplifies a polymorphic microsatellite repeat. Because of the technical advantages of using an indel polymorphism for quantitative genotyping [36], and because of the close linkage to nga280, we employed MN1.2 for the quantitative genetic analyses of the pBCs.

MN markers were designed by identifying short insertions or deletions between Col-0 and Wa-1 present in the sequence database provided by the Magnus Nordborg laboratory (http://walnut.usc.edu/apache2-default) [62]. The sequence and modification of these primers was summarized previously [36]. Forward primers for markers nga280, F5I14, and nga692 [24,63] were labeled with 6-FAM, NED, and ROX respectively.

For the pBC populations, chromosome doses were inferred from quantitative fluorescent PCR as previously described [36]. Individuals were categorized depending on their chromosome number. Individuals with 10, 15, or 20 chromosomes were classified as euploids, while all other individuals were classified as aneuploid. Individuals from the CCWW *F*_2_ population were only partially karyotyped as data were only available for four of the five chromosome types. Individuals for which all quantitative genotypes were consistent with tetraploidy (*n* = 72) were classified as euploid, while individuals for which the quantitative genotypes for at least one chromosome type indicated the presence of additional or missing chromosomal copies were classified as aneuploid (*n* = 18).

### Statistical genetic analyses.

For each population, the numbers of Col-0 and Wa-1 alleles were counted. For example, a CWWW genotype contributed three Wa-1 alleles and one Col-0 allele. The ratio of C to W within the population was compared to the expected 1:1 ratio using chi-squared tests. Transmission ratio distortion in the CCWW *F*_2_ as compared to the expected 1:1 ratio was tested by chi-square analysis. Test significance was set at a *p*-value < 0.0083, equivalent to a Bonferroni correction for a *p* < 0.05 and six independent tests on four chromosome types. Only the euploid individuals were used for this analysis (*n* = 72), to eliminate any possible effect of aneuploidy on genotype.

In order to statistically test the effect of marker genotypes on various traits, genotypes were expressed quantitatively as the percentage of Wa-1 allele. These values were inferred directly from the genotypes determined using quantitative fluorescent PCR. For example, the CCCW genotype was assigned a quantitative genotype value of 25. These values were used to test the relationship between marker genotype and ASI (see below) by regression analysis. For the CWW *F*_2_ populations, regressions associated with *p*-values < 0.01 were considered significant, equivalent to a Bonferroni corrected *p* < 0.05 for five independent tests on three chromosome types. Similarly, for the pBC populations, regressions associated with *p*-values < 0.005 to control for ten independent tests on ten chromosome types.

### ASI.

A value for ASI was calculated for each genome content class of the CWW *F*_2_ population. The expected frequency of each genome-content class was calculated assuming random assortment of three sets of chromosomes and no selection for or against karyotypes. For each genome content class, the observed frequency was calculated by dividing the number of individuals observed in that genome content class by the total number of individuals. The values for ASI for each genome content class were obtained using the following formula: ASI = log 2 (expected frequency/observed frequency). Using this formula, chromosome number classes that were overrepresented relative to their expected frequencies were assigned negative ASI values, while positive ASI values indicated selection against a chromosome number class. Some of the genome content classes included few individuals. We tested an alternative version of ASI in which these classes were excluded from the analysis. Although the *p*-values obtained were higher, the percentage of Wa-1 allele was significantly affected by ASI at the same markers as presented in Table 1.

A similar approach was applied to the pBC populations with the exception that genome content classes were replaced by chromosome number classes since each of the pBC individuals had been molecularly karyotyped [36]. ASI values were calculated separately for each pBC population.

## Supporting Information

### Accession Numbers

The Arabidopsis Information Resource (TAIR) (http://www.arabidopsis.org) accession number for Wa-1 is CS6885.

## Abbreviations

4x-Col: tetraploid Col-0
ASI: aneuploidy selection index
Col-0: diploid A. thaliana ecotype Columbia
pBC: pseudo-backcross
RIL: recombinant inbred line
*SDI*: *SENSITIVE TO DOSAGE IMBALANCE*
Wa-1: naturally occurring tetraploid A. thaliana ecotype Warschau-1
